# Supplementary material for: A mathematical model of potassium homeostasis: Effect of feedforward and feedback controls
Source: PLoS Comput Biol. 2022 Dec 20;18(12):e1010607. doi: 10.1371/journal.pcbi.1010607 (PMC9812337; doi:10.1371/journal.pcbi.1010607)
Supplement: S1 Text — (PDF) [file pcbi.1010607.s001.pdf]

# A mathematical model of potassium homeostasis: Effect of feedforward and feedback controls

Melissa M. Stadt[1*], Jessica Leete[2], Sophia Devinyak[3], and Anita T. Layton[1,4,5,6]

**1** Department of Applied Mathematics, University of Waterloo, Waterloo, ON, Canada
**2** Computational Biology and Bioinformatics Program, Duke University, Durham, NC, USA
**3** Department of Physics and Astronomy, University of Waterloo, Waterloo, ON, Canada
**4** Cheriton School of Computer Science, University of Waterloo, Waterloo, ON, Canada
**5** Department of Biology, University of Waterloo, Waterloo, ON, Canada
**6** Department of Pharmacy, University of Waterloo, Waterloo, ON, Canada

* Corresponding Author: mstadt@uwaterloo.ca

# Supporting Information

## Data availability

All code files used to generate the results presented in the manuscript are available at https://github.com/Layton-Lab/Kregulation.

## Effect of feedback and feedforward control mechanisms

We conducted additional simulations to investigate the effects of the individual control mechanisms. This study is analogous to those shown in Fig. 7 of the manuscript, however instead of showing the impact of showing only one feedforward or feedforward control mechanism, we started by turning all the mechanisms off and then include one control mechanism on at a time to see the impact on plasma [$K^+$] and intracellar [$K^+$]. The results are shown in Fig. S1.

## Response to repeated $K^+$ loading

The simulation results for Fig. 8 on the model's response to repeated $K^+$ loading for various muscle-kidney cross talk mechanisms is shown in Fig. S2 for days 2-6 where $K^+$ loading occurs. This shows more clearly the impact on renal $K^+$ handling during this period.

## Impact of parameters on muscle-kidney cross talk during $K^+$ loading and depletion

To investigate the impact of the parameter $m_{\mathrm{Kic}}$ in Eq. 28 from the manuscript on muscle-kidney cross talk we conducted simulations analogous to the $K^+$ loading and depletion experiments shown in manuscript Fig. 8 and 9, respectively. We conducted simulations for varied values of $m_{\mathrm{Kic}}$ using the muscle-kidney cross talk mechanism that targets distal tubule $K^+$ secretion (i.e., Case MKX-DT-sec). The results for loading and depletion are shown in Fig. S3 and S4, respectively. Notably, when we decreased $m_{\mathrm{Kic}}$ by 50%, we can see that indeed muscle-kidney cross talk has a large impact on the

predicted intracellular and plasma $K^+$ concentrations in both the loading and depletion experiments. This shows how muscle-kidney cross talk likely plays a key role in ensuring that intracellular $K^+$ concentration returns to baseline after a period of $K^+$ loading or depletion without significantly altering the plasma $K^+$ concentration.
