# Supplementary figures and images for: A mathematical model of potassium homeostasis: Effect of feedforward and feedback controls
Source: PLoS Comput Biol. 2022 Dec 20;18(12):e1010607. doi: 10.1371/journal.pcbi.1010607 (PMC9812337; doi:10.1371/journal.pcbi.1010607)

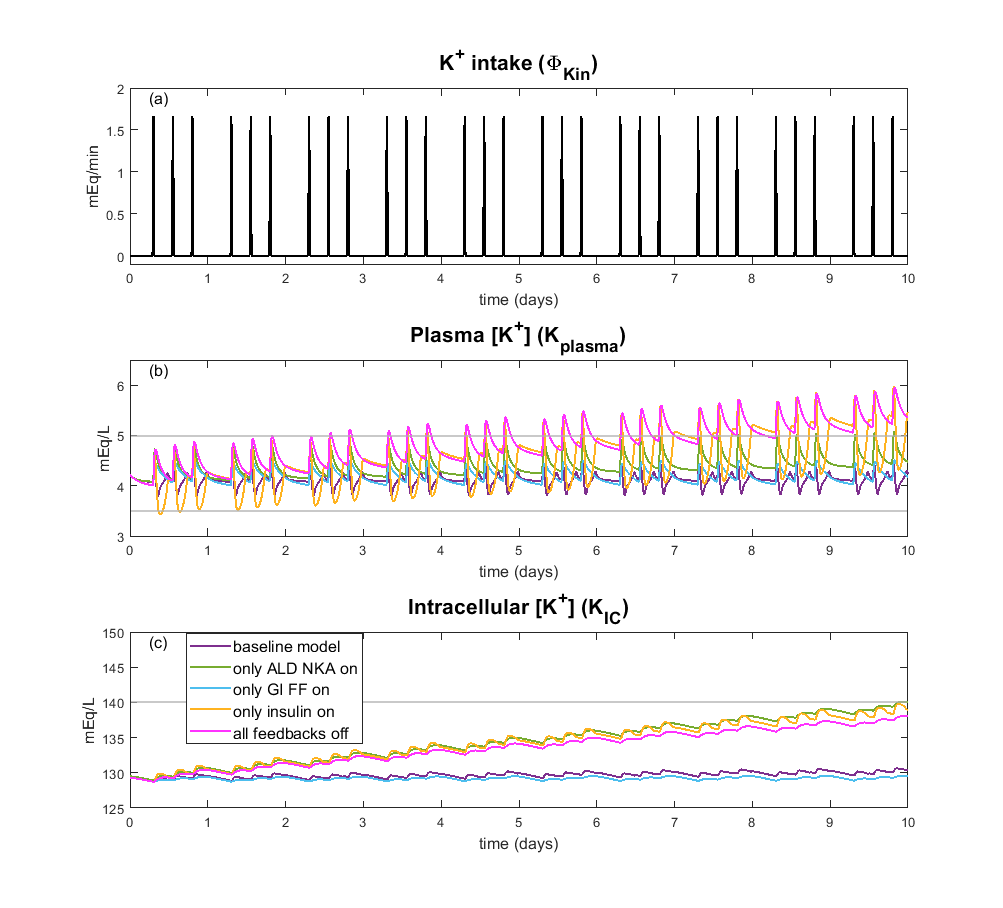

Supplement: S1 Fig — Simulation results for plasma K+ concentration (B) and intracellular K+ concentration (C) for 10 days of normal K+ intake ((A);100 mEq/day, 3 typical meals) for the baseline model, all control mechanisms off except the ALD effect on Na+-K+-ATPase uptake (ρal), all control mechanisms off except the gastrointestinal feedforward mechanism, all control mechanisms off except insulin (ρinsulin), and baseline model with all feedback effects off. Grey lines indicate normal range for Kplasma and KIC. (TIF) [file pcbi.1010607.s002.tif]

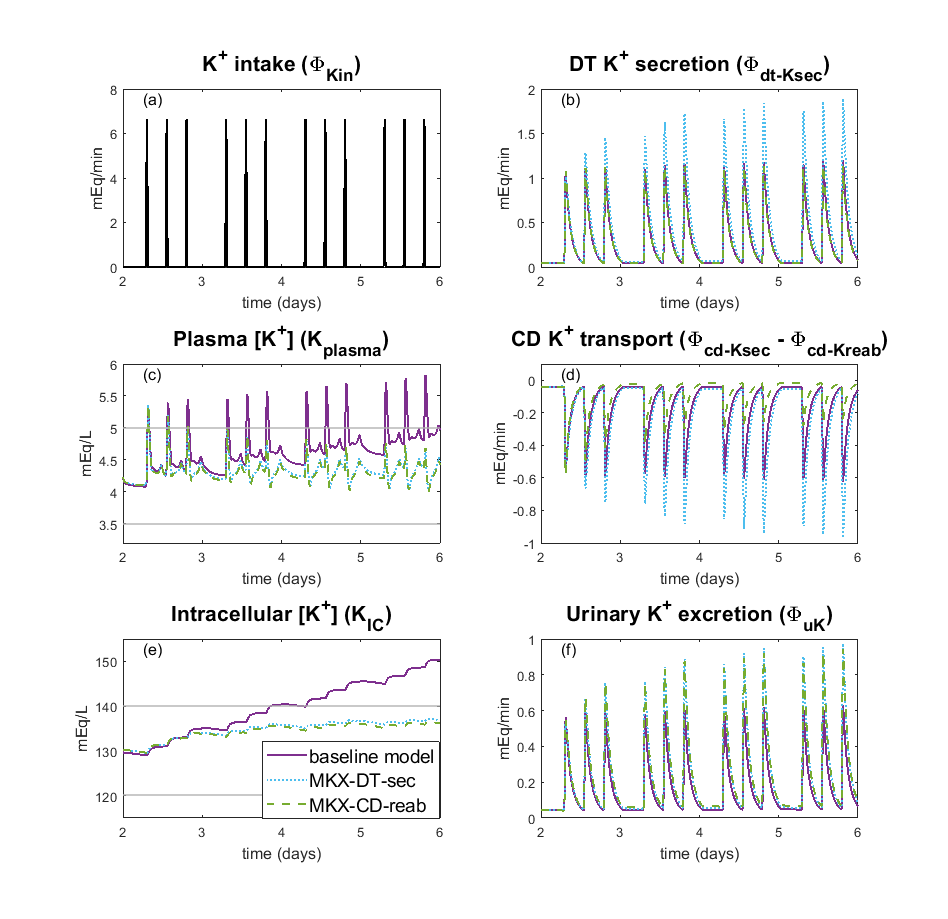

Supplement: S2 Fig — The same simulation results as shown in Fig 8 of manuscript. Simulation results for K+ loading experiments for the baseline model and muscle-kidney cross talk simulations (Case MKX-DT-sec and Case MKX-CD-reab). Potassium intake (A) is the same for all three simulation types. Note that Case MKX-CD-sec is not plotted since it had little impact from the baseline model results. Horizontal grey line shows normal range for plasma K+ concentration (C) and intracellular K+ concentration (E). CD: collecting duct, DT: distal tubule. (TIF) [file pcbi.1010607.s003.tif]

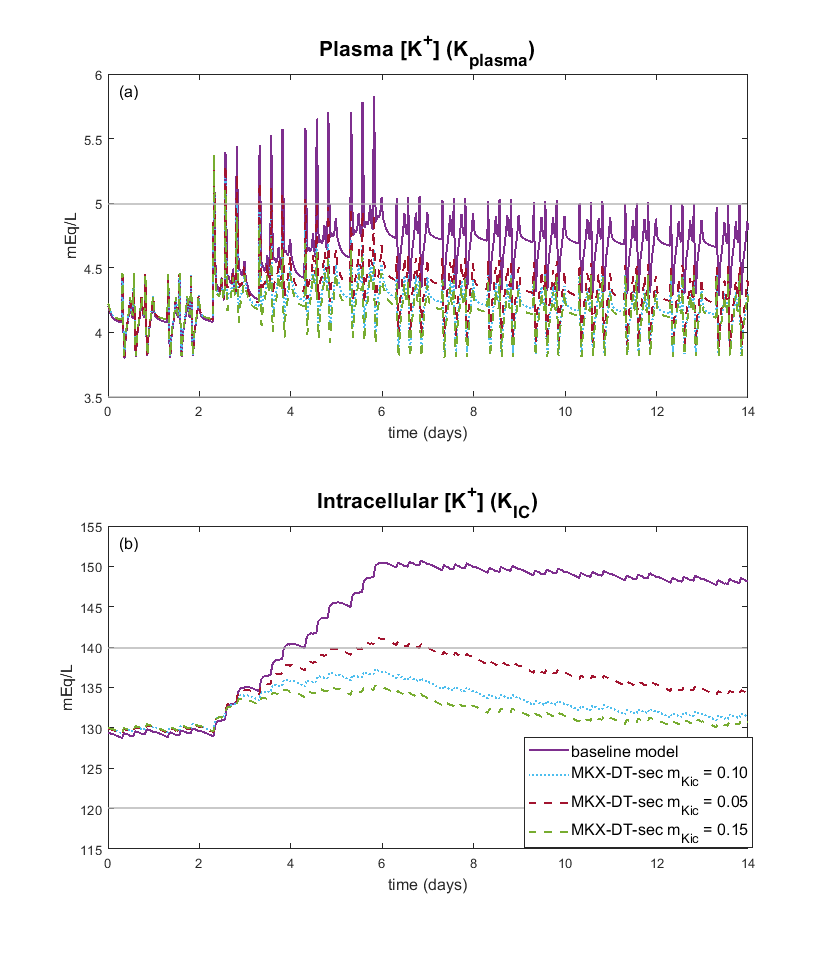

Supplement: S3 Fig — Simulation results for K+ loading experiments for the baseline model and distal tubule K+ secretion muscle-kidney cross talk simulations (Case MKX-DT-sec) for varied values of the parameter mKic. Potassium intake is the same for all simulation types. Horizontal grey line shows normal range for plasma K+ concentration (A) and intracellular K+ concentration (B). (TIF) [file pcbi.1010607.s004.tif]

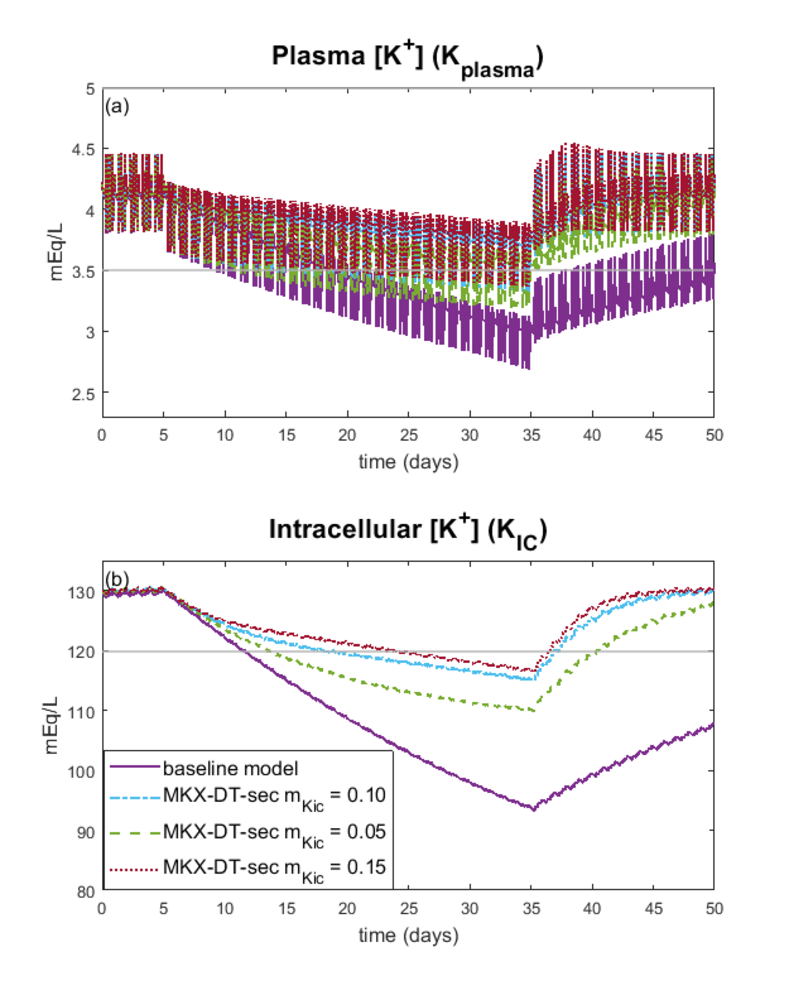

Supplement: S4 Fig — Simulation results for K+ depletion experiments for the baseline model and distal tubule K+ secretion muscle-kidney cross talk simulations (Case MKX-DT-sec) for varied values of the parameter mKic. Potassium intake is the same for all simulation types. Horizontal grey line shows normal range for plasma K+ concentration (A) and intracellular K+ concentration (B). (TIF) [file pcbi.1010607.s005.tif]
