# Supplementary material for: A mathematical model of potassium homeostasis: Effect of feedforward and feedback controls
Source: PLoS Comput Biol. 2022 Dec 20;18(12):e1010607. doi: 10.1371/journal.pcbi.1010607 (PMC9812337; doi:10.1371/journal.pcbi.1010607)
Supplement: Attachment — Submitted filename: ReviewerResponse.docx [file pcbi.1010607.s006.docx]

Reviewer #1: In this study the authors describe a compartmental model of whole-body K+ regulation, and investigate the effect of various regulatory mechanisms on K+ homeostasis. The manuscript is well structured and clearly written. Moreover, I compliment the authors on the very interesting work, establishing a model of great value providing nice theoretical insights on a putative mechanism (e.g. muscle kidney crosstalk) that could contribute to K+ homeostasis under high K+ loading or depletion. However, my main concern is that some of the predictions may be dependent on the modeling assumptions and parameter values (which is always the case so not a problem per se), which are currently not shown. The sensitivity analysis is only done on the baseline model, not on the cases with(out) particular regulatory mechanisms and/or extreme K+ loading/depletion. As such, in addition to some more concrete question below, I think showing the parameter sensitivity to those simulation results as well would benefit the manuscript.

We appreciate the reviewer’s thorough response and recommendations for improvements on our study. Indeed, parameter and modeling assumptions is often a difficulty in developing mathematical models of complicated physiological systems such as K^+^ regulation. We believe that the revisions made and addition of a supporting information section will improve our study.

Questions/comments:

Are there K+ related pathologies/diseases? Did/could you explore those with the model?

There are plenty of K^+^-related diseases. Hypokalemia and hyperkalemia are common electrolyte disorders caused by changes in K^+^ intake, altered excretion, or transcellular shifts. Diuretic use and gastrointestinal losses are common causes of hypokalemia, whereas kidney disease, hyperglycemia, and medication use are common causes of hyperkalemia. Both hypo- and hyperkalemia} can be potentially fatal. The focus on the present study is on normal K^+^ physiology. While K^+^ pathophysiology is definitely interesting, it is a whole entire new ballgame---it involves much more than adding a couple more simulations. As such, we would rather leave that to a future study.

Figure 1: if you add 2/3 + 25%+3%+10% > 100%?
I do not understand? Consider specifying a range per segment.

We changed lower bound for the loop of Henle to 20% based on Sufit & Jamison 1983 PMID: 6638178.

Out of curiosity – how does the gastrointestinal feedforward control mechanism work? (mechanistically) through which signaling networks is the gut linked to the kidney? Same for the putative muscle-kidney crosstalk. Could you elaborate/speculate?

A few experimental studies have provided evidence that the splanchnic area is the location for the GI K^+^ sensor (Rabinowitz et al. 1977, 1988; Morita et al. 2000; Tsuchiya et al. 2004). What the signal is appears to be less well understood. Oh et al. 2011 examined and ruled out a number of GI hormones. Sorensen et al. 2013 examined and ruled out aldosterone. Glucagon is also unlikely. El Moghrabi et al. 2010 showed, in mice, that renal tissue kallikrein can lead to an increase in K^+^ excretion in concert with tissue kallikrein excretion following a single meal. The investigators further showed that in tissue kallikrein knockout mice, there was a greater increase in serum K^+^ following a meal. Thus, although renal tissue kallikrein is not a GI-derived signal, it could play a role in this phenomenon at the level of the kidney.

The muscle-kidney crosstalk is hypothetical; its mechanism and signalling pathway are what we imagine them to be.

How were the parameters determined? Are these unique solutions? Could similar results be obtained with other (yet unidentified) feedback mechanisms? If so, how certain can we be of the current results?

The baseline parameters for the renal compartment, other than the fitting parameters specified in the “Model Parameters” section, were largely determined from reports summarized in Greenlee et al. 2019 as well as predictions by Layton & Layton 2019 for segmental K^+^ transport predictions. The remaining parameters were chosen by fitting data from Preston et al. 2015 to a single meal experiment using the interior-point optimization algorithm.

Indeed, it is possible there are other (unknown) feedback mechanisms, but our baseline model reflects what is currently known in the literature and could be updated and expanded upon to include more mechanisms as the knowledge of K^+^ regulation mechanisms expands.

Fig 2 – for completeness: add GFR and ALD explanation to the caption

Done.

“As the filtrate passes through the nephrons, K+ (and other solutes and fluid not
represented in this model) is reabsorbed and returned to the general circulation along some tubular segments, and secreted into the lumen along the distal segments.” Since K+ is an ion & since some transporters are e.g. co-transporters; should the other solutes (or some of them) not also be considered; as they will also influence the K+ transport in the kidney?

The reviewer is correct that K^+^ transport is coupled to other solutes, either directly via a co-transporter or directly via membrane potential. But for simplicity, the transport of other solutes and fluid is not represented in this model. This is now clarified in the manuscript.

What are γal × γKin in equation 14 – consider referring in the text to the aldosterone paragraph and equations 23-24. (similar for ρal × ρinsulin – link them better to the equations later on).

The references to the specific equations were added.

Why multiply these effects and not add them?

The effects are multiplied because they represent a stimulation/inhibition of distal tubule K^+^ secretion or NKATPase rather than an additional source of secretion or intracellular potassium uptake.

“ We capture this effect by the scaling factor ρal (from Eq. 8), based on findings by Phakdeekitcharoen et al. [23].” Consider elaborating this more - on what is this fitted? What are the limitations/assumptions of these data (and thus the fit)?

This was added into the “Model Parameters” section to elaborate on how this part of the model was fit to the data presented in this study. Some limitations to this approach are that this is only one study with a small cohort of patients. However, this is the only study who (to our best knowledge and extensive search) that has measured the impact of ALD on NKATPase uptake. As such, we were able to capture physiological trends in K^+^ balance.

“Rosic et al. [27] found that percent maximal Na+-K+-ATPase stimulation is a function of serum insulin concentration.” To help the reader the assumptions/limitations of this fit would be useful to add.

This sentence has been revised since it is misleading. Rosic et al. 1985 did show how insulin effects NKATPase uptake in isolated cells (and insulin impact on NKATPase uptake has been accepted by scientist who study K^+^ homeostasis, e.g., see McDonough et al. 2022, Youn et al 2011), however we did not fit the equation directly to the Rosic study and rather used fitting parameters to fit to data from Preston et al 2015. This is described in the “Model parameters” section.

Where can we see the model fit? How well does the model fit the data?
Consider adding these figures to the supplement.

The model was fit so that steady state concentrations were in normal range and to plasma concentration data from Preston et al. 2015 for a single meal under varied conditions. This is described in the “Model parameters” section and are shown in Fig. 4f.

Table 2 – consider adding a column with measured experimental values, to see the how good the model matches reality.

We added Table 3 to the manuscript with reported ranges and their references for the K+ content in various compartments.

It is not clear what the exact initial conditions are of Meal only”, “KCl only”, or “Meal + KCl”. Please clarify (e.g. with a table in the supplement).

The initial conditions are the steady state values listed in Table 2. We added clarification to the figure captions with references to Table 2 to make this clear to readers.

Figure 4D – The model seems to underestimate the Kplasma for KCI only and overestimate for meal only – why is that? Which mechanisms might be missing in the model that could explaint his? Similarly for the urinary excretion (figure 5D) – the match for simulation results with the experimental data of the meal with KCl does not match well? Could you comment?

For both of these cases, it is likely more renal mechanisms that are not fully being captured in this model. One of the major limitations is that we only represent K^+^ and the influence of K^+^ in this model, rather than other electrolytes and fluid volume. It is well known that Na^+^ and K^+^ play key roles and interact with one another, but to include these mechanisms would require a significantly more complicated model.

The feedforward loop (green) has an important influence in intracellular K+ (figure 7) – how dependent is this result on parameter values?

We ran simulations analogous to the protocol in Fig 7 (10 days of 3 equal meals for 100 mEq/day) for the baseline model and then with the parameter A_Kin (from Eq. 27, GI FF effect) varied for +/-10% and more. Model predictions were not significantly changed in this parameter range.

Figure 7 – it would be interesting to see how this figure is different in pathological states (so how the different feedback systems contribute (maybe worsen?) to K+ control.

As noted above, the focus of this study is on K^+^ balance in normal physiology. Disease states are complicated --- everything changes! And in all likelihood, the feedback systems changes too (the set point, feedback gain). Without incorporating all those differences, which is beyond the scope of this work, any comparison will not be very meaningful.

Figure 7 – how do you explain that the order of the cases is not the same for plasma K+ and intracellular K+? E.g. for plasma the pink is highest, whereas for intracellular K+ the green is highest? Also – how come that the green line (only GI FF off) is higher than when all mechanisms are off (pink one)? Please clarify in the text.

This is indeed an interesting observation. It has to do with the competing effects of the multiple feedback and feedforward control mechanisms. In the GI FF effect off only (green) in Fig 7, there are still the effects of insulin and ALD on NKATPase uptake which brings more K^+^ into the intracellular fluid. In the “all feedbacks off” (pink) these mechanisms are off, so the stimulation of NKATPase from insulin and ALD do not occur, therefore less K^+^ being moved into the intracellular fluid. Since intracellular K^+^ fluid content can greatly impact plasma K^+^ concentration this is why we see such an increase in plasma K^+^ concentration in the “all feedbacks off” case.

Your question inspired us to add a figure to the supplementary information section that shows the effect of having just one of the control mechanisms on. This shows how intracellular K^+^ content is increased when the insulin and ALD impact on NKATPase is added. We found this type of simulation helpful in our analysis, but chose not to include it in the main manuscript as we felt it took away from the main message because it is a bit confusing to think through, but would indeed help readers who seek more information.

Figure 8 – it would be good to have magnifications in the supplement (e.g. of a representative peak) – it is currently not easy to see the differences between the cases.

We added figures to the supplement where the results are shown for days 2-6 so that it is more clear what is happening during the K^+^ loading period.

It would be good to elaborate the findings of the muscle-kidney crosstalk mechanisms, in particular, how do the parameters thereof influence the outcome, e.g. the omega?

We varied the parameter m_Kic in the muscle-kidney cross talk equation (Eq. 28) by +/- 50% to see the impact on the results for K^+^ loading and depletion in muscle-kidney cross talk. These results were added to the supporting information document. We can see that model trends are similar for the different cross talk parameters, but rather changes how fast the intracellular and plasma K^+^ concentrations return to baseline levels. Even with the decrease of 50%, we can see that the intracellular K^+^ concentrations quickly return to physiological ranges after K^+^ loading without significantly increasing plasma K^+^ concentration. Similar results were found for K^+^ depletion.

Case MXK-DT-sec reduces distal tubule K+ secretion to almost 0 – is this physiological? By which transporters does this secretion occur? How would this affect the secretion of other solutes?

Depending on K^+^ flow, the distal segments are capable to K^+^ secretion or reabsorption. At sufficiently high K^+^ flow, K^+^ secretion occurs through the ROMK and BK channels. At low K^+^ flow, the BK channels are closed, and any K^+^ flux would occur through the ROMK channels only. K^+^ fluxes via BK and ROMK would generate a current and affect the membrane potential. As such, changes in K^+^ fluxes would impact other apical ion channels and non-electroneutral membrane transporters (e.g., ENaC) as well as the paracellular ion fluxes.

“Pettit and Vick [31] ... in patients with hyperaldosteronism.”
This paragraph would maybe fit better in the introduction as it describes the state of the art. Please elaborate what was learned from these models & which aspects were not modelled (the research gap) and which ones you addressed here?
If you prefer to leave it in the discussion, then I would extend the paragraph by comparing the predictions of these models with those presented in the manuscript and hypothesize on potential discrepancies.

As suggested by the Reviewer, we moved this section to the Introduction and addressed the research gap.

The muscle kidney crosstalk is still hypothesized – what do we learn from the modeling predictions that can be used to do design some experiments to explore the muscle kidney crosstalk more/confirm it?

Model simulation results suggest that under loading or depletion this signal is essential for returning to normal baseline models, consistent with experimental observations by Rabelink et al. (1990) that after loading or depletion K^+^ levels return quite quickly. As for experiments that can be done to interrogate this hypothetical pathway---that is a challenging question, given that the mechanism for this crosstalk is still vague. Possibly, a panel of mediators that could communicate signals between the muscles and the kidneys can be investigated, i.e., turned off individually to assess any impact on K^+^ response.

“ There are other mechanisms that impact K+ homeostasis that have not been captured in the models presented in this study. One such mechanism is circadian rhythms . . . Progesterone has also shown to play a role in K+ regulation in both males and females [41, 42].”
Could these mechanisms make that muscle-kidney crosstalk is not necessary (to help maintaining K+ balance)?

We cannot say for certain, but our guess is that these mechanisms (circadian rhythms, progesterone) do not rule out the need for the hypothesized muscle-kidney crosstalk. A major role of that crosstalk is for the kidney to respond to changes in K^+^ balance without a significant change in plasma K^+^ concentration. It isn’t clear to us how these other mechanisms could have done that job.

The discussion nicely lists some future perspectives – I would encourage the authors to specify what is needed to take those steps (is it just a matter of doing it or do we need more data, if so how to get that data etc)?

We thank the Reviewer for their kind words. We have expanded the perspectives section to included what needs to be done for these future extensions.

Reviewer #2: Well conceived project and well written manuscript.
A few minor corrections.
Typos and language:
Abstract, line 6: "homeostasis" instead of "regulation"?

Fixed.
Only outside Abstract, line 10: "K+ excretion" is typed twice..

Fixed.
Change last sentence of Summary: The significance of our "model is that it allows" to ......K+ "homeostasis" ..

Changed.
Introduction, line 30: delete "in"

Deleted.
Internal K+ balance, line 84: missing "is" before "excreted".

Added “is”
For consistency use "mEq" throughout the MS, instead of switching between mmol and mEq.

Changed all instances of mmol to mEq.

Reviewer #3: The authors have developed a novel compartmental model of whole-body K+ regulation to explore specific feedforward and feedback effects on K+ homeostasis. The authors present in this manuscript the use of this model to explore the impact of various feedback mechanisms under several perturbations to gain preliminary insight into the significance of muscle-kidney cross talk. The assumptions and limitations of the developed model are clearly described and discussed in the manuscript and the authors suggest several areas ripe for future study with a suitable extensions to the presented model. As such, the presented model sets the baseline that can serve as the basis for a variety of future studies while still being able to elucidate preliminary insights to guide future studies.

The manuscript is very well written and clearly describes each of the simulation protocols used in the presented model analysis. It is great to see the authors putting a lot of effort into ensuring physical units are comprehensively presented throughout the model, although I did struggle with the mix of concentrations in mEq/L, ng/L, ng/dl, pmol/L, etc... - while I suspect that is just cause I am not used to seeing the Eq units, it would be nice to use consistent units throughout.

We found inconsistencies in our units and made changes accordingly.

The authors should be commended for making their model implementation available on Github and I was very happy to be able to reproduce simulation results presented in the manuscript - thanks so much for providing a README that guides users through the various Matlab files. I would recommend that the authors tag the version of the code used to generate the specific results presented in the manuscript to ensure that future readers are able to unambiguously get to that version of the implementation. This could be achieved with a release on Github or publishing a version to zenodo or figshare or similar services. Also, given that this model could provide the basis for reuse in many future extensions, it would be good to be clear about the reusability of the code by adding a license under which the implementation is being made available on Github.

We thank the Reviewer for the great suggestion. We changed our README on Github so that it is clearer which scripts to run to generate original figures from the manuscript. We created a version release to keep in mind future updates to the model to make it clear which version we used in this specific study. This is a great tip. We will also look into other possible services to upload our code for more access after publication.

Minor revisions/suggestions

Line 360 - extra '.' after the Preston citation.

Removed.

Line 469 - "100 mEq per da" should probably be "...per day"

Corrected.

Line 502 - misplaced '('

Revised.

Lines 525-531 - MXK should probably be MKX and DTT should be DT ?

Corrected.

Figure 9 caption - consistency in the use of "cross talk" vs "crosstalk"

Changed all instances of “crosstalk” to “cross talk”.
