# Supplementary material for: A mathematical model of potassium homeostasis: Effect of feedforward and feedback controls
Source: PLoS Comput Biol. 2022 Dec 20;18(12):e1010607. doi: 10.1371/journal.pcbi.1010607 (PMC9812337; doi:10.1371/journal.pcbi.1010607)
Supplement: Attachment — Submitted filename: ReviewerResponse.docx [file pcbi.1010607.s007.docx]

There were no reviewer comments in the decision with the title “Decision from PLOS Computational Biology (PCOMPBIOL-D-22-01420R1) - [EMID:98048339891a92f3]”. As such we do not have any reviewer responses.

We list here the changes made in this resubmission:

- Checked that all figures were compliant using the PACE digital diagnostic tool and uploaded the new figures.
- Simulation data and code is available at <https://github.com/Layton-Lab/Kregulation>
  - This information is added into the supporting information
- Fixed supplementary information figure numbering
